# Image Correlation Between Digitally Reconstructed Radiographs, C-arm Fluoroscopic Radiographs, and X-ray: A Phantom Study

**DOI:** 10.7759/cureus.51868

**Published:** 2024-01-08

**Authors:** Sebastian Wangler, Janic Hofmann, Helen L Moser, Michael Kuenzler, Rainer J Egli, Michael Schaer

**Affiliations:** 1 Orthopaedic Surgery and Traumatology, Inselspital, Bern University Hospital, University of Bern, Bern, CHE; 2 Diagnostic, Interventional, and Paediatric Radiology, Inselspital, Bern University Hospital, University of Bern, Bern, CHE

**Keywords:** ct, phantom study, drr, c-arm, digitally reconstructed radiographs

## Abstract

Objective: Digitally reconstructed radiographs (DRRs) are planar two-dimensional (2D) X-rays derived from a three-dimensional (3D) computed tomography (CT) dataset. DRRs allow the simulation of radiographs of all desired views and facilitate preoperative planning. However, orthopedic surgeons rely on C-arm fluoroscopic imaging during surgery to verify fracture reduction and implant placement. Pincushion distortion represents a technical limitation of fluoroscopic imaging, resulting in a greater distance between points at the periphery of the image compared to the center. This project, therefore, aimed to assess the image correlation between digitally reconstructed radiographs (DRRs) and fluoroscopic imaging (C-arm) using conventional radiographs (X-ray) as a control.

Methods: A 3D-printed cubic prototype and an anatomical humerus bone model were used. C-arm fluoroscopic radiographs and conventional X-ray images were taken in an anteroposterior (AP) view at 10-degree steps while rotating the objects from 0 to 90 degrees. CT scans were made and used to compute and export DRRs in AP view at 10-degree rotational steps from 0 to 90 degrees. The surface area (cm^2^) was measured and compared between the different modalities. For automated image analysis of the anatomical humerus model, matching (%) between modalities was calculated using the structural similarity index (SSIM).

Results: The overall regression was statistically significant in all models, with an R^2^ >0.99 when comparing all three imaging modalities of the prototype. Surface correlation in the anatomical humerus model was R^2^ 0.99 between X-ray and C-arm and R^2^ 0.95 between C-arm and X-ray to DRRs, respectively. The SSIM was highest for comparing DRR and C-arm images (0.84±0.01%).

Conclusions: The study indicates a strong agreement between digitally reconstructed radiographs and X-ray/C-arm images. DRRs, therefore, represent a valuable tool for research and clinical application.

## Introduction

Digitally reconstructed radiographs (DRRs) are planar two-dimensional (2D) X-rays derived from a three-dimensional (3D) computed tomography (CT) dataset. The planning and application of radiotherapy were one of the first clinical implementations of DRRs [1-3]. Here, the correct patient positioning can be verified by comparing a DRR from the planning stage with a portal image captured by the accelerator in the treatment stage [4]. Concerning osseous structures, DRRs have helped identify new anatomical landmarks for intraoperative orientation during the first metatarsal osteotomy in hallux valgus deformity correction. Computing DRRs from the first metatarsal bone in different planes allowed a better understanding of the association between metatarsal rotation in the axial plane and the resulting changes in metatarsal head image morphology [5]. DRRs also offer an excellent opportunity to evaluate the impact of the radiographic viewing perspective on the reproducibility of numeric measurements. In doing so, it has been observed that a deviation of the scapular anteversion of only five degrees results in a false measurement (>2 degrees) of the critical shoulder angle (CSA) when compared to a true anteroposterior (AP) view [6].

The literature suggests that DRRs are a valuable tool for evaluating osseous structures. Moreover, numeric measurements on DRRs are interchangeable with conventional X-ray imaging. However, orthopedic surgeons often rely on C-arm fluoroscopic imaging to verify fracture reduction and implant placement during surgery. A technical limitation of C-arm fluoroscopic imaging is pincushion distortion, which results in a greater distance between points at the image's periphery than at the center [7]. Therefore, the present project aims to investigate if pincushion distortion limits the comparability between fluoroscopic C-arm images and DRRs, using a 3D-printed cubic prototype and an anatomical humerus bone model, with conventional radiographs (X-ray) used as a control.

## Materials and methods

Materials

A custom 3D-printed cubic prototype and an anatomical humerus bone model (Synbone©, ref. 5150) were used. Two holes with different diameters were integrated on both sides of the cubic prototype to control its positioning in all planes (leveled position = even distance between the two superimposed outlines of the circles). The prototype was 3D printed (Ultimaker S3) using acrylonitrile butadiene styrene (ABS) plastic filament as previously described [8].

Imaging procedure

**Figure 1:**
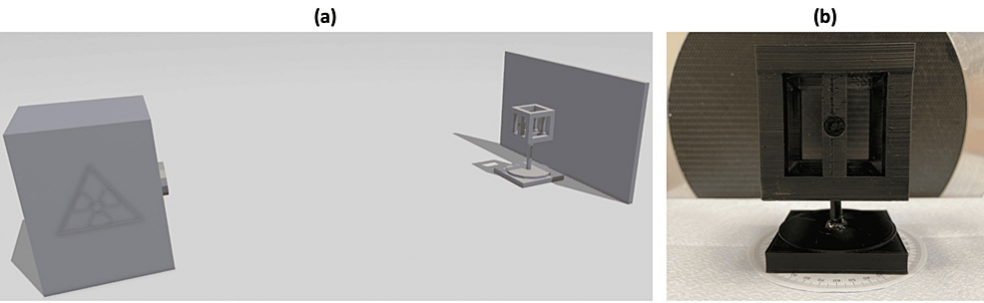
(a) A scheme showing the experimental setup with the 3D-printed prototype on a rotating platform 65 cm away from the radiation source (C-arm, X-ray). (b) 3D-printed prototype placed in front of the C-arm.

**Figure 2:**
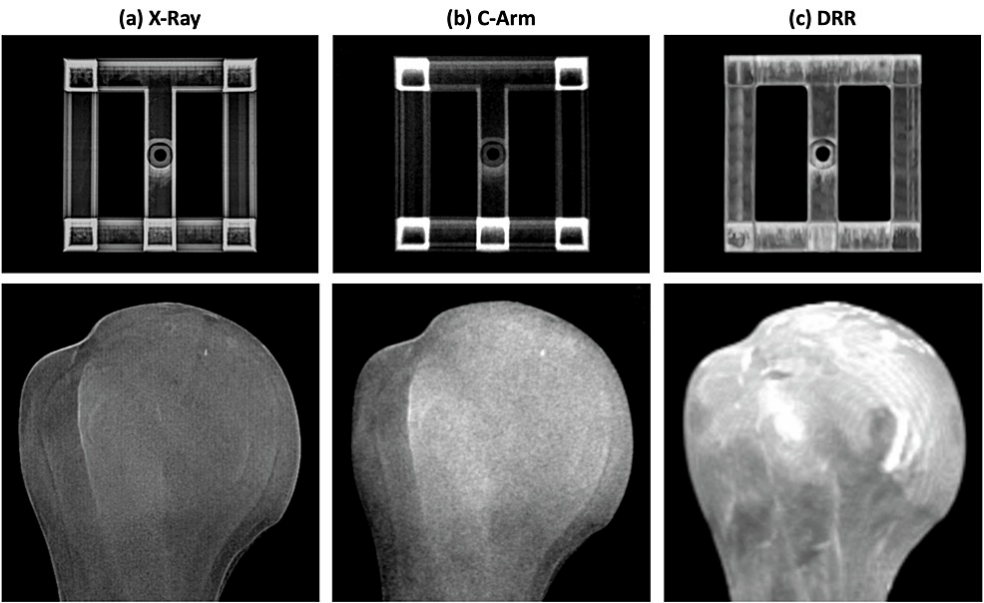
Treated images of the 3D printed prototype (upper raw) and the anatomical humerus bone model (lower row) used for analysis. (a) X-ray image, (b) C-arm fluoroscopic image, (c) digitally reconstructed radiography (DRR). The total surface area was calculated in cm^2^.

The prototype and humerus bone model were attached to a custom-made rotating platform. The platform allowed for precise object rotation in 10-degree steps. C-arm fluoroscopic radiographs (Ziehm Vision C-arm) and conventional X-ray images were taken while rotating the object from 0 to 90 degrees (Figure 1). This led to ten anteroposterior (AP) view images for the prototype and the humerus bone model, respectively (Figure 2a,b shows an example at 0° rotation). The distance between the radiation source and the prototype or humerus bone model was constant at 65 cm (Figure 1). CT scans (TOSHIBA AQUILION CXL 128) of the prototype and humerus bone model were made. Based on CT data, AP-view DRRs were computed using Horos v.3.3.6. The 3D-printed prototype and the humerus model were then artificially rotated in 10-degree steps from 0 to 90 degrees. The AP-view DRR was exported for each step, leading to ten images for the prototype and the humerus bone model, respectively (Figure 2c shows an example at 0° rotation).

Analysis

Two independent observers measured the object's surface area in cm^2^ (Horos v.3.3.6). The surface area was compared among the three imaging modalities using linear regression models (Prism v9). For automated analysis of the humerus bone model, matching (%) between images among the different modalities (DRR, C-arm, X-ray) was calculated using the structural similarity index (SSIM, structural_sim, Anaconda Platform, Spyder v5.1.5). The SSIM values range between 0 and 1, where 1 refers to a 100% match between the compared images. The mean ± SD of percentage-matching between X-ray/DRR, C-arm/DRR, and X-ray/C-arm is reported. An intra-modality comparison between DRR/DRR was used to verify the method.

## Results

**Figure 3:**
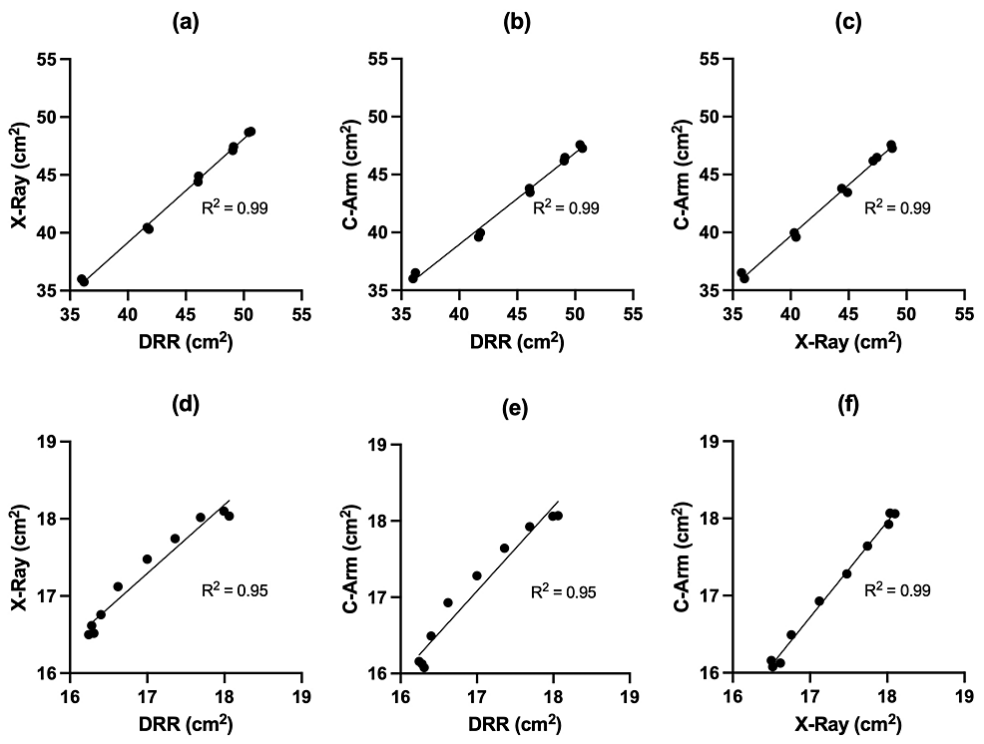
A comparison of surface measurement in cm2 in pictures taken at the same degree of object rotation among the different imaging modalities (C-arm/X-ray/DRR). The line represents the fit of the linear regression model. (a-c) 3D printed prototype, (d-f) anatomical humerus model. DRR: digitally reconstructed radiograph.

Simple linear regression was used to assess the similarity between the different imaging modalities based on surface measurement in cm² for the prototype and humerus model, respectively. R^2^ represents a measure that determines the proportion of variance in the dependent variable that can be explained by the independent variable. The overall regression was statistically significant in all models with an R^2^ >0.99 when comparing all three imaging modalities of the 3D-printed prototype (Figure 3 a-c). Surface correlation in the anatomical humerus model was R^2^ 0.95 between X-ray and DRRs, R^2^ 0.95 between C-arm and DRRs, and R^2^ 0.99 between C-arm and X-ray, respectively (Figure 3 d-f). The SSIM of the humerus model calculated between the different modalities is reported as follows: DRR/DRR: 100±0%, C-arm/DRR: 0.84±0.01%, X-ray/DRR: 0.77±0.01%, C-arm/X-ray: 0.80±0.02%.

## Discussion

In preoperative planning, DRRs offer an excellent opportunity to simulate any desired conventional view of a displaced fracture. However, during surgery, fracture visualization occurs with fluoroscopic C-arm imaging. Pincushion distortion is a technical limitation of fluoroscopic imaging, resulting in a magnification effect towards the image's periphery as the lens magnification increases with axial distance. In clinical applications, this can lead to the misplacement of guidewires as the image intensifier does not reflect the intended trajectory. If guidewires are aimed from the image periphery, the distortion can result in misplacement in the range of 1 cm [9]. Similarly, fluoroscopic pelvic images taken during total hip replacement are associated with vertical distortion up to 2 cm at the periphery of fluoroscopic images. This can lead to unintended implant positioning and limb-length discrepancies [10]. It is crucial to understand the extent to which DRRs, used for preoperative planning, can be translated and compared to intraoperative fluoroscopic imaging.

The present study does not indicate that pincushion distortion limits the comparability of C-arm images to DRR when the object is placed in the center of the image. The current study indicates a significant correlation between DRR, C-arm imaging (3D printed prototype: R^2^ 0.99, humerus: R^2^ 0.95), and X-rays (3D printed prototype: R^2^ 0.99, humerus: R^2^ 0.95). Concerning the comparison of DRR and X-ray images, the results of the present study align with the findings of other authors. The intra- and interrater reliability of standardized measurements in DRRs computed from foot CTs and the corresponding conventional X-rays is excellent [11]. Similarly, the correlation coefficient of the alpha angle, a two-dimensional (2D) radiographic measure of femoral head sphericity, between radiographs and DRRs has been shown to be excellent [12].

Regarding the automated image comparison (SSIM), the highest matching was found when comparing C-arm images to DRRs (C-arm/DRR 0.84±0.01%). However, the contrast of the X-ray images used in this study differs from the DRRs and the C-arm images (Figure 2). The SSIM compares the images' luminance, contrast, and structure. Several limitations of the application of the SSIM on medical images have been reported. They include uniform pooling, distortion underestimation near hard edges, and instabilities in regions of low variance and insensitivity in areas of high intensities [13]. Therefore, the lower contrast in the X-ray images may influence SSIM calculation, which could explain the lower percentage-matching for the comparisons including X-ray images (X-ray/DRR 0.77±0.01%, C-arm/X-ray 0.80±0.02%). Nevertheless, real-time comparison of DRRs and intraoperatively acquired C-arm images might represent an interesting clinical application. In the first step, fracture reduction or implant positioning could be preoperatively planned on the CT dataset. In the second step, the DRRs of the planning could be exported, including all possible conventional image projections. Lastly, C-arm images acquired during surgery could be automatically registered to the respective exported DRR. The identified DRR could then be superimposed on the C-arm monitor to facilitate fracture reduction or implant positioning.

According to the present study, C-arm fluoroscopy can be compared to DRRs. Therefore, surgeons can preoperatively identify the optimal visualization angles and use these views as blueprints for intraoperative fluoroscopic imaging. This might help to quickly identify the desired view and, through that, reduce surgeons' intraoperative radiation exposure [14]. The comparability might be further improved by using cross-modality image-to-image translation, as described in the comparison of DRRs and conventional X-rays [15].

This study has several limitations. First, a humerus saw-bone model does not include soft tissue, potentially altering the image quality. Secondly, only one C-arm model was included in this study; results might vary depending on the model type. Thirdly, for normalization purposes, the distance between the radiation source and the object was held constant at 65 cm. Changing the distance between the source and the object leads to a magnification effect, which might influence the comparability to DRRs. Lastly, the current study represents a phantom experiment. Further research is required to investigate whether the findings of this study also apply to patient-derived images.

## Conclusions

The study indicates a strong agreement between digitally reconstructed radiographs (DRRs) and X-ray/C-arm images. Therefore, DRRs used in the preoperative planning of orthopedic approaches can be compared to intraoperative fluoroscopic C-arm imaging, which helps quickly identify the desired view and reduces radiation exposure. Future applications might involve software-based image comparison, automatically registering preoperative DRRs to C-arm images acquired during surgery to verify planned fracture reduction or implant positioning.
